# Formation of Microcapsules of Pullulan by Emulsion Template Mechanism: Evaluation as Vitamin C Delivery Systems

**DOI:** 10.3390/gels10060355

**Published:** 2024-05-21

**Authors:** Esther Santamaría, Naroa Lizarreta, Susana Vílchez, Carme González, Alicia Maestro

**Affiliations:** 1 Chemical Engineering and Analytical Chemistry Department, Faculty of Chemistry, Universitat de Barcelona, Marti i Franques, 1, 08028 Barcelona, Spain; 2 INSA-UB, Nutrition and Food Safety Research Institute, University of Barcelona, 08921 Santa Coloma de Gramenet, Spain; 3 Institute of Advanced Chemistry of Catalonia, Consejo Superior de Investigaciones Científicas (IQAC-CSIC) and Networking Research Center on Bioengineering, Biomaterials and Nanomedicine (CIBER-BBN), Jordi Girona, 18-26, 08034 Barcelona, Spain

**Keywords:** pullulan, emulsion encapsulation template, microcapsules, vitamin C, release kinetics, oxidation protection

## Abstract

Pullulan is a polysaccharide that has attracted the attention of scientists in recent times as a former of edible films. On the other hand, its use for the preparation of hydrogels needs more study, as well as the formation of pullulan microcapsules as active ingredient release systems for the food industry. Due to the slow gelation kinetics of pullulan with sodium trimetaphosphate (STMP), capsules cannot be formed through the conventional method of dropping into a solution of the gelling agent, as with other polysaccharides, since the pullulan chains migrate to the medium before the capsules can form by gelation. Pullulan microcapsules have been obtained by using inverse water-in-oil emulsions as templates. The emulsion that acts as a template has been characterized by monitoring its stability and by optical microscopy, and the size of the emulsion droplets has been correlated with the size of the microcapsules obtained, demonstrating that it is a good technique for their production. Although some flocs of droplets form, these remain dispersed during the gelation process and two capsule size distributions are obtained: those of the non-flocculated droplets and the flocculated droplets. The microcapsules have been evaluated as vitamin C release systems, showing zero-order release kinetics for acidic pH and Fickian mechanism for neutral pH. On the other hand, the microcapsules offer good protection of vitamin C against oxidation during an evaluation period of 14 days.

## 1. Introduction

Some biopolymers can be produced by a variety of microorganisms and act as soluble gums, providing special physicochemical properties [1]. Due to these properties, numerous applications have been found, mainly in the food industry [2] and the cosmetics industry [3]. Among the most common applications are emulsifiers, stabilizers, gelling agents, binders, edible film formers, etc. Advances in different technologies allow for the modification of these biopolymers in order to offer them to the consumer in different ways for the desired applications. The most used biopolymers in the food industry are polysaccharides and proteins like pectins, alginates, gellan, xanthan gum, gelatins, and chitosan, as examples [2,4,5,6]. Alginates and pectins have a fairly simple gelation mechanism based on the egg box mechanism induced by the presence of divalent cations, normally provided by calcium salts, which allows rapid gelation of the biopolymer, allowing gels to be obtained in bulk form [7,8,9], macrospheres [10,11], or even according to microsphere techniques [12,13,14,15]. Moreover, chitosan presents slower gelation kinetics when genipin is used as a cross-linking agent [16,17]. One of the biopolymers that has been attracting the attention of scientists in recent years is pullulan. The structure of pullulan was first described by Bender in 1959 [18], although pullulan itself was first reported a year earlier by Bernier [19], who isolated the pullulan form from *Aureobasidium pullulans*. It consists of maltose units connected by α (1→4) glycosidic bonds, whereas consecutive maltotriose units are connected to each other by α (1→6) glycosidic linkages, providing a molecular formula (C_6_H_12_O_5_)_n_. Pullulan has been especially studied in the biomedical field [20,21,22,23] due to its nonimmunogenic, nontoxic, biocompatible, and inert nature.

In the food field, the growing interest in pullulan is fundamentally due to its ability to form biodegradable films, which can be used as a substitute for commercial plastics or as edible films to wrap all types of foods. The most extensive work is being developed for the protection of fruits and vegetables [24,25,26] due to the great transparency and vegetable origin of pullulan films, which makes them ideal for consumption by vegans, unlike films that result from the use of chitosan [27,28].

In the United States, the FDA accepted pullulan as a safe compound in 2002. In the European Union, it was recently accepted as a food additive (E 1204) for capsules, tablets, and films under directive 2006/52/EC. The use of pullulan in the food industry is also allowed in some Asian countries, Russia, and some South American countries [29].

The use of pullulan as a matrix gel for the encapsulation of active ingredients is limited to the formation of bulk gels because until now it has not been possible to form pullulan capsules in the same way that other polysaccharides with the capacity to form gels using divalent cations as cross-linking agents. The conventional way to form these capsules is the dropping of a polysaccharide solution into a solution of the cross-linking agent, so that gelation is quickly triggered once the drop enters contact with the divalent ions. The gelation of pullulan in the presence of sodium trimethaphosphate has been reported [30,31] (Figure S1), but its gelation kinetics is very slow as described by Santamaría et al. [32]. Due to the slow gelation, capsules cannot be formed through the dropping method, since the pullulan chains migrate to the medium before the capsules can form by gelation.

As described in previous works [32], the gelation time of pullulan with STMP depends on the concentration of pullulan used to form the hydrogel. Santamaría et al. [32] determined that for pullulan concentrations of 10%, 8% and 6% *w*/*w* the gelation time varied from approximately 10 min, 30 min, and 60 min, respectively. These times are too long to obtain beads such as those that can be obtained using polysaccharides that have gelation mechanisms through the “egg box” mechanism.

Some works have described the possibility of forming biopolymer microcapsules by using emulsions as templates [33,34]. Larger microcapsules generally provide better protection than smaller ones due to their less exposed surfaces, but exhibit poor dispersion in the final food product, while smaller diameter microcapsules have low microencapsulation efficiency [35]. Thus, it seems appropriate to use a compromise size, which needs to be controlled. By using the emulsion template technique for microencapsulation, the droplet size can be controlled by the mixing rate and the type and % of surfactants [36]. To the best of our knowledge, the encapsulation of hydrophilic compounds into pullulan beads has not been reported.

The purpose of this study is to obtain pullulan microbeads using the emulsion template method in order to encapsulate active ingredients and to evaluate their behavior as controlled release systems of molecules of special interest for the food industry. Because the polymer that forms the hydrogel is in the aqueous phase of the emulsion, in this case, water-in-oil inverse emulsion, so that the droplets of the aqueous phase can be kept separated and thus the gel can be formed in the shape of spheres of active ingredients that can be encapsulated, must all be hydrophilic to be mixed with the pullulan.

In this case, the encapsulated compound is a hydrophilic model compound such as vitamin C in order to evaluate the suitability of the microcapsules obtained to be incorporated into different foods. It has been decided to use vitamin C as an active ingredient because it is a vitamin of high interest in the food industry. Its hydrophilic character allows it to be easily mixed with pullulan in the aqueous phase for subsequent encapsulation and due to its nature, its preservation is important given the ease with which it oxidizes and loses its beneficial properties for health.

## 2. Results and Discussion

### 2.1. Gelation Time

**Figure 1:**
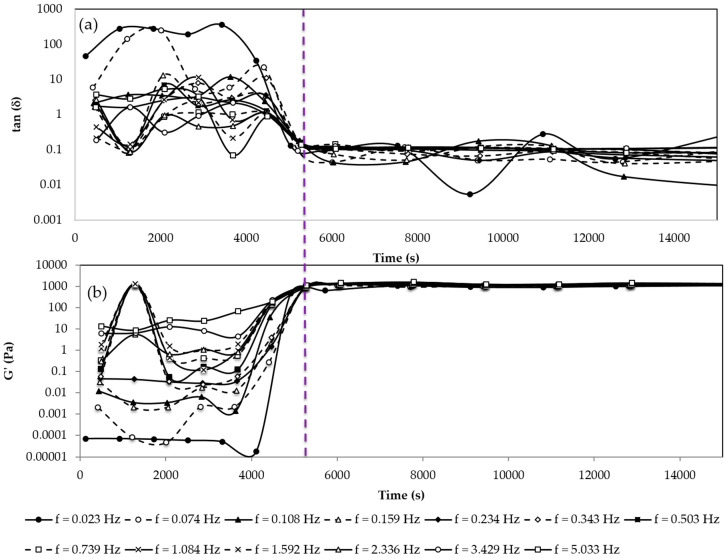
(**a**) Tan δ at different frequencies vs. time of gelation. (**b**) Elastic modulus (G′) at different frequencies vs. time of gelation. For all experiments pullulan 4% *w*/*w*, KOH 1.7% *w*/*w*, and STMP 1% *w*/*w*. T = 25 °C.

An important factor to take into account when working with systems that transform from a solution to a gel is the determination of the gelation time. It is defined as the time at which the cross-linking reaction between the biopolymer and the cross-linking agent extends to the whole bulk and confers to the sample properties of a soft solid, i.e., the sample does not flow under small stress and it has a more similar behavior to that of a solid than that of a liquid. Over this time, further hardening can occur. Typically, gelation points are detected using the Winter–Chambon criterion [37,38,39,40,41] which postulates that at this juncture, the tan δ (the ratio between storage modulus, G′, and loss modulus, G″) versus time tends to have very low values and becomes independent of the frequency. This is due to the much higher values of G′ (elastic modulus) compared to G″ (viscous modulus) that determine the primarily solid characteristics of the sample. The gelation time of the dispersed phase used in emulsion-templated gelation was determined by preparing a sample with the composition of this phase and carrying out frequency sweeps over time, monitoring viscoelastic functions. Figure 1a shows the variation of the tan δ = G″/G′ for this dispersed phase used later during the microcapsule formation process, for all the frequencies used.

According to the Winter and Chambon method, in order to determine the gelation time, different oscillatory frequency sweeps must be carried out from the moment of sample preparation until the end of the gelation process. For each viscoelasticity experiment, the values of tan δ and G′ at each frequency are recorded. Then the values obtained at the same frequency for all the oscillatory frequency sweeps are represented as a function of time (Figure 1a).

**Figure 2:**
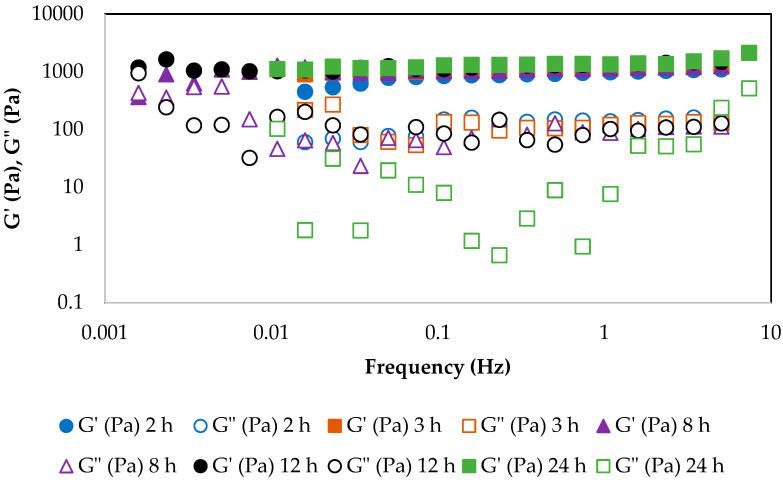
Evolution of elastic modulus (G′) and viscous modulus (G″) at different frequencies with time. For all experiments, pullulan 4% *w*/*w*, KOH 1.7% *w*/*w*, and STMP 1% *w*/*w*. T = 25 °C.

It can be observed that around a time of 5200 s, the values of the tan δ converge and tend toward zero, showing that from approximately this value, the pullulan is already cross-linked, extending the network to the whole bulk of the sample. Figure 1b shows the evolution of the elastic modulus with time for each frequency. It is observed that for the same time value of 5200 s the values of G′ are relatively independent of the frequency and very high, around 1100 Pa. In this way, it can be assumed that after 2 h of the reaction the gel has been formed. After the gelation time is reached, a mild increase in viscoelastic parameters is observed, up to around 24 h, due to further formation of some cross-links (Figure 2). For other ratios of pullulan/STMP, the evolution of the gel after the gelation time is stronger [32]. In the present case, most of the cross-links have been produced at the gelation time, but these completely develop in the following hours. It can be seen that the elastic modulus increases with time, going from values of 1100 Pa at the gelation time to values of 1250 Pa after 8 h, 1450 Pa after 12 h, and 2120 Pa after 24 h. These changes in the value of G′ show that although it can be considered that the bonds created between the pullulan chains by the STMP molecules are all already established, the gel hardens over time until the structure is completely developed.

For this reason, the emulsions used as templates for microcapsules formation were left stirring for 24 h in order to ensure that the emulsion drops resulted in completely gelled beads.

### 2.2. Emulsion and Microcapsules Characterization

**Figure 3:**
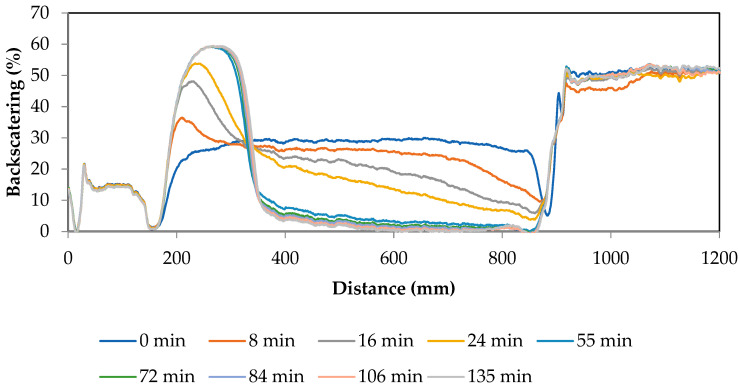
Evolution of the emulsion backscattering with time: 20% *w*/*w* dispersed phase (4% *w*/*w* pullulan, 1.7% *w*/*w* KOH, 1% *w*/*w* STMP), 10% surfactant (S80/T80 = 51/49).

The stability of the emulsion was evaluated by monitoring the evolution of the backscattering of the emulsion with time. Figure 3 shows its evolution during the first 2 h. It can be seen how the backscattering decreases in the upper part of the tube, and increases in the lower part, indicating that there is a destabilization mechanism due to sedimentation of the dispersed phase. It should be noted that during the gelation process of pullulan in emulsion, the emulsion is kept agitated and, therefore, the droplets-beads are maintained in dispersion, and in the backscattering test the emulsion is maintained without agitation.

**Figure 4:**
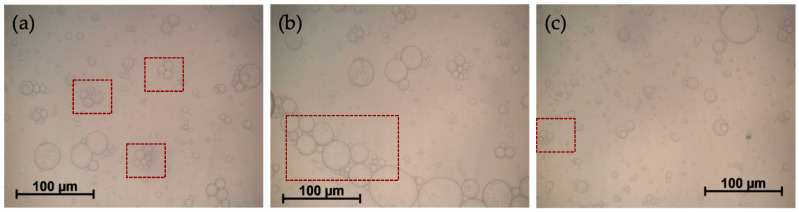
Microscopy images of the different emulsion regions (**a**–**c**) used as a template to obtain capsules. 20% *w*/*w* dispersed phase (4% *w*/*w* pullulan, 1.7% *w*/*w* KOH, 1% *w*/*w* STMP), 10% surfactant (S80 = 51/T80 = 49).

The droplet size of the emulsion was characterized in order to elucidate if there was a correlation between the droplet size of the emulsion and the size of the microcapsules obtained, in such a way that the emulsion droplet size could determine, as a template, the size of the microbeads obtained. Different micrographs of the emulsion are shown in Figure 4a–c. It can be seen that droplets are quite polydisperse. It can also be seen how in many cases the droplets are agglomerated but without losing their identity (red boxes). These flocculated droplets would be the first ones to reach sedimentation and destabilize the emulsion, as observed in Figure 3. Despite the flocculation of the droplets, when the characterization of the emulsion was carried out by microscopy, they were counted as independent droplets, because they did not lose their identity.

**Figure 5:**
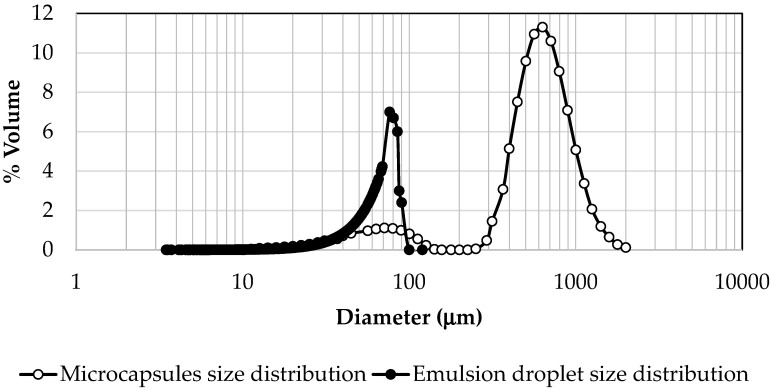
Comparison of the droplet sizes of the emulsion and the microcapsules obtained.

In order to obtain the size distribution of the emulsion, 1000 drops were measured and the distribution was represented in Figure 5 showing the % volume provided by each diameter. The average Brouckere diameter, d_4,3_, of the emulsion droplets is 55 μm ± 6 μm. The size distribution of microbeads, measured by laser diffraction, is plotted in the same figure (Figure 5).

**Figure 6:**
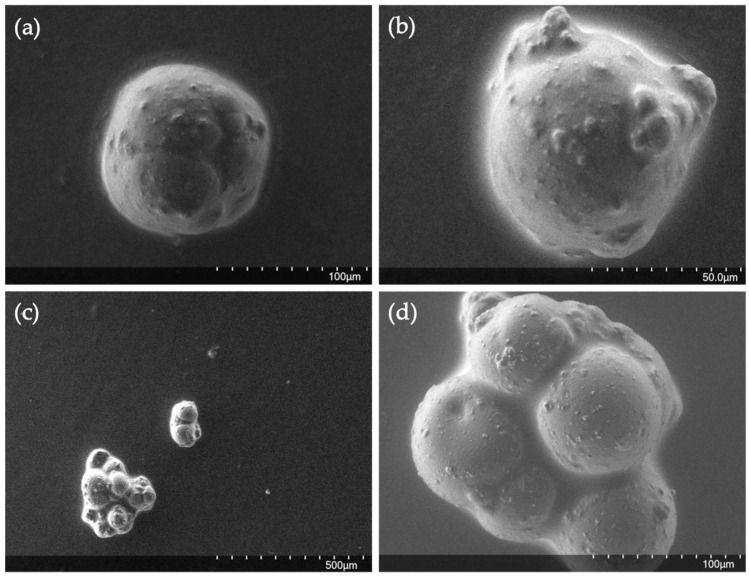
Different SEM images of the microcapsules obtained (**a**–**d**). (4% *w*/*w* pullulan, 1.7% *w*/*w* KOH, 1% *w*/*w* STMP).

It can be observed that the microcapsules present two size distributions, one of them in the range of 20–100 μm, which coincides with the distribution obtained for the emulsion droplets (3–90 μm), while the other is a distribution of much larger sizes (200–900 μm). As previously mentioned, the emulsion, which acted as a template maintaining the dispersed phase in the form of drops for the time necessary to carry out the gelation reaction, suffers some flocculation during the first two hours, causing agglomeration of a part of the droplets. It causes the formation of the second peak of the size distribution of the microcapsules, derived from the gelation of the flocculated drops. When the microcapsules are observed by SEM (Figure 6a–c), it can be seen that some of them (Figure 6a,b) have a spherical shape, similar to the original emulsion drops, while in Figure 6c,d capsules appear that have a series of protuberances. These protuberances have a certain spherical shape and look like independent drops that have gelled all together, glued with each other, as a result of the flocculated droplets present in emulsion (Figure 4 inside red boxes). In this way, the drops that remained isolated produced microcapsules with the same diameters as those recorded in the emulsion (distribution of 10–90 μm) and the agglomerated drops resulted, when gelled, in the second peak of the size distribution, which could not be obtained when the emulsion droplets were measured because they were counted as independent droplets of each other.

In this way, it can be stated that the gelation technique of the emulsion template presents good results since the pullulan microcapsules can actually be obtained from the gelation of the dispersed phase of the emulsion. Emulsion template gelation has previously been used mainly to obtain alginate microcapsules using a water-in-oil emulsion, where the alginate solution is the dispersed phase. Gelation is then induced by further addition of oil containing CaCl_2_ nanoparticles (external gelation) [42], or the addition of acetic acid when an insoluble salt of calcium is already present in the dispersed phase (internal gelation). In this case, the acetic acid migrates to the dispersed phase and dissolves the salt, inducing gelation [34,43].

Lin et al. [44] studied the differences between internal and external gelation of alginate in emulsions. They concluded that external gelation produced smaller bead diameters, but in both cases, the beads obtained were larger than the emulsion droplets. A similar result was reported by Ribeiro et al. [45], who prepared alginate–chitosan microspheres through internal gelation. The mean diameter of the emulsion droplets was around 200 μm, and the microspheres obtained had a mean diameter of 250–1000 μm, quite similar to the microbeads obtained in the present work. The microspheres obtained in the present study could be assimilated to an internal gelation since the cross-linking agent STMP is already present in the dispersed phase, and any component is not further added for gelation, as it just needs enough time to be produced. Therefore, in this case, the emulsion gelation technique is used to provide time for chain cross-linking to occur, avoiding the dissolution of droplets before gelation occurs, as it happens when the method of dropping in the CaCl_2_ bath is used.

### 2.3. Vitamin C Release

The microencapsulation efficiency has been determined to be 70 ± 2%. There are several mathematical models to describe the release of drugs or active ingredients in the food industry. One of the most used is the Korsmeyer–Peppas [46], which describes the release of the active ingredient through polymeric systems and establishes an exponential relationship between the amount of active ingredient released and time [47]. In addition, the Korsmeyer–Peppas equation classifies the release profiles according to whether they conform to a first or a zero order. To put it mildly, when the release exponent, *n*, is around 0.43–0.45, the system is classified as Fickian and it is correlated with a release driven by a first-order equation. If *n* ≥ 1, the model is non-Fickian and it is known as super case type II diffusion, and it correlates with a release of the active ingredient of zero order if n exponent has a value near 1, which is governed by forces such as swelling and/or relaxation of the polymer chain. In the event that the exponent *n* is between 0.5 and 1, the release is determined by a combination of diffusion and swelling. It is important to note that the Korsmeyer–Peppas model is only valid for $M_{t}$/$M_{\infty }$ values lower than 0.60 and cannot be applied to higher values that fail to meet the underlying assumptions used to derive the equation [47,48].

**Figure 7:**
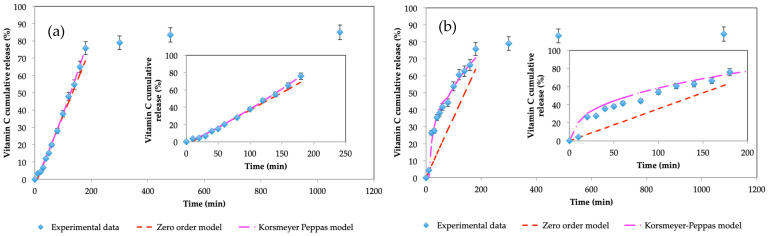
Vitamin C release profiles from microcapsules in (**a**) pH = 1.8 and (**b**) pH = 7.0.

Figure 7 shows the release profiles of vitamin C in two different media: an acidic medium (Figure 7a) and a neutral medium (Figure 7b). It can be observed that in both cases, the final amount of vitamin C released is very similar and nearly 80% of the encapsulated vitamin C is released across 180 min. The significant difference in both release curves occurs in the shape of the curves for the first ~180 min and the value of *n*. For an acidic release medium, the Korsmeyer–Peppas equation fits well, providing a value of *n* of 1.18. It is therefore indicative that it is a transport process linked to super case type II diffusion. On the other hand, when the release in a neutral medium is evaluated, the Korsmeyer–Peppas equation also presents a good fit but with an *n* value of 0.40, very close to a mechanism governed by Fickian diffusion, as shown in Table 1.

According to these results, it could be assumed that when the capsules are in an acidic environment they suffer from some type of degradation and swelling that leads to the constant release of vitamin C [49], but which is slower than in a neutral environment. Other authors reported that when hydrogels suffer from some type of micromechanical damage, this can induce an increase in the swelling of the polymeric matrix [32,44,50]. To form the network of cross-linked pullulan chains, the presence of a basic medium is needed, in this case promoted by the addition of KOH to the dispersed phase of the emulsion, in order to create the appropriate conditions for the pullulan to cross-link [30,31]. The presence of the acidic medium may alter the pullulan chains and the cross-linking with STMP molecules may be affected. If the polymeric network degrades, it becomes weaker, the flow of water that enters the microcapsules can increase, and it dilutes the active compound, reducing the driving force responsible for diffusion by Fick’s law.

Previous studies [32] did not show that bulk pullulan gels deteriorated in the presence of acidic pH, in the case of the microcapsules obtained, as they are smaller than the bulk any micro damage can suffer greater grind down or erosion that leads to a matrix deterioration.

For the case of pH = 7, the behavior of the microcapsules changes and presents the most frequent Fickian mechanism of matrices formed by hydrogels described by other authors [51,52,53]. This fact shows the capacity of pullulan gels to be pH-responsive and, depending on their composition and the relationship between the concentration of pullulan and KOH, to be able to change the type of release mechanism to adapt it to the needs of release according to the active ingredient used.

### 2.4. Shelf Life Assessment of Vitamin C

The protection against oxidation that pullulan microbeads offered to encapsulated vitamin C was evaluated by analyzing its oxidation degree over time by iodometry. To make the control samples, free vitamin C was used and the degradation of vitamin C and encapsulated vitamin C over time were compared. All the samples were exposed to daylight at T = 25 ± 2 °C

**Figure 8:**
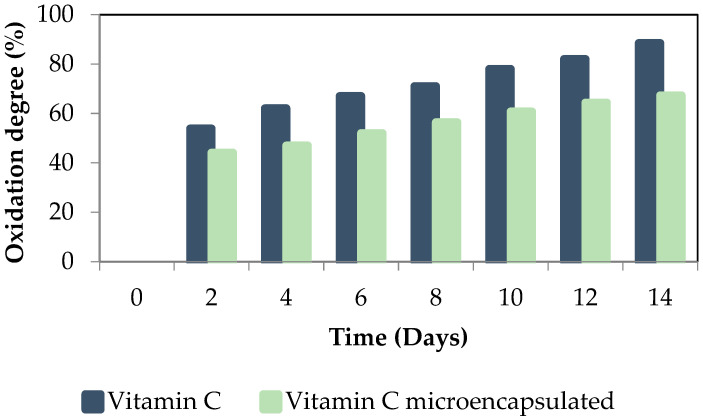
Encapsulated and non-encapsulated vitamin C oxidation degree across 14 days of storage time.

As shown in Figure 8, the shelf life of vitamin C encapsulated with pullulan microcapsules was higher than that of free vitamin C, as the oxidation degree was reduced by around 20% through encapsulation. Vitamin C is very sensitive to light, oxygen, and temperature. No protection against light is offered by pullulan beads, as pullulan gels are very transparent. In any case, the oxidation was retarded, probably because there was no direct contact with oxygen. Other authors [54,55] successfully encapsulated vitamin C and checked the effectiveness of chitosan nanoparticles in protecting vitamin C. In their case, the protection against oxidation was 10% better than that presented in the current work. However, the chitosan gels are not as transparent as pullulan ones, and some protection against light when concerning chitosan beads could explain this difference. Nevertheless, the results obtained are similar to those reported by Comunian et al. [56] who investigated the protection against oxidation of microcapsules formed by gelatin and agar gum as matrices.

## 3. Conclusions

The emulsion template methodology was successfully used to prepare pullulan microcapsules. The emulsion acts as a template and the microspheres present the same size distribution pattern as the emulsion droplets. There is a second size distribution because the emulsion presents a flocculation of droplets, therefore forming larger microcapsules resulting from the union of droplets gelled together, as observed by SEM. The capsules obtained were evaluated as drug delivery systems. It was observed that in the case of the release of vitamin C in an acidic medium, the release mechanism is of zero order, related to swelling due to the weakening of the gel network, presumably because the gelation method implies that the pullulan is in a basic medium for the cross-linking of the chains, and when acidifying the material it deteriorates. On the contrary, at a neutral pH, the release mechanism follows the Korsmeyer–Peppas model with an *n* value indicating a release of the drug near Fickian diffusion. On the other hand, the protective capacity of the capsules against the oxidation of vitamin C was studied, showing good stability in the pullulan microcapsules.

## 4. Materials and Methods

### 4.1. Materials

Pullulan, M_w_ = 373 kDa, M_n_ = 100 kDa, from ITV reagents was used. Potassium hydroxide pellets at 85% purity (06103), sodium trimetaphosphate (STMP) (T5508), synthetic non-ionic surfactant Tween 80 (P1754), Span 80 (S6760), starch (03967), and vitamin C acid (A5960) were purchased from Sigma-Aldrich, Spain. Sunflower oil (Borges Brand) was purchased from a local supermarket. Potassium iodide (A3172) purity ≥ 99.5% and iodine (131771) purity ≥ 99.8% were purchased from Panreac. MilliQ water was used without further purification.

### 4.2. Molecular Weight Determination

The weight-average molar mass (M_w_), number-average molar mass (M_n_), and polydispersity index (M_w_/M_n_) of pullulan were determined following the procedure used by Morales–Martínez et al. [57] with some modifications. A high-performance size-exclusion chromatography (HPSEC) was carried out in a Water Alliance 2695 HPLC system coupled with a Waters 2414 refractive index detector. Chromatographic separation was performed on two columns (PSS Suprema Analytical Ultrahigh + PSS Suprema Analytical Linear S, both of 10 μm, 8 × 300 mm) at 30 °C. The mobile phase was 0.2 M NaNO_3_ + 0.01 M NaH_2_PO_4_, pH = 7, with 0.02 g/100 g sodium azide (NaN_3_) as an antimicrobial agent. The samples (2 mg/mL) dissolved in the mobile phase and filtered through 0.45 mm nylon membranes were injected into the HPLC system. The injection volume was 100 mL, and the flow rate of the mobile phase was 0.8 mL/min. A calibration curve of ten pullulans standards from 500 to 739,000 Da was constructed using Empower 3 software from Waters. The characterization was carried out in the unit from Serveis Científico-Tècnics of Universitat de Barcelona (CCiT-UB). The molecular weight of M_w_ = 373 kDa and (M_w_/M_n_) = 3.7.

### 4.3. Emulsion Formation

The formation of the emulsion was performed according to the method described by Lupo et al. [34] with some modifications. The water-in-oil emulsion was prepared by magnetic stirring at 200 rpm with temperature control at 25 °C. The continuous phase (oil phase) was prepared mixing sunflower oil (70% *w*/*w*) with 10% *w*/*w* of Span 80 (S80) and Tween 80 (T80) surfactant mixture in a ratio 51/49. On the other hand, the dispersed phase (aqueous phase) was prepared by dissolving 4% *w*/*w* pullulan in water and subsequently basifying it with (KOH) = 1.7% *w*/*w* in another beaker. Once the pullulan was dissolved, 0.1% *w*/*w* of the vitamin C was added The STMP (1% *w*/*w*) as described by Santamaría et al. [32] was added. Once the dispersed phase was prepared, it was added drop by drop using a pipette to the oil-–surfactant mixture. The emulsion was continuously stirred for 24 h to ensure complete cross-linking of the pullulan molecules.

### 4.4. Microcapsules Obtaining

After 24 h, the emulsion was vacuum filtered. Once the capsules were retained, they were washed three times with 50 mL of water each. The washing waters were separated in order to determine the encapsulation efficiency.

### 4.5. Encapsulation Efficiency

In order to evaluate the encapsulation efficiency, the filtered solution and the three washes were collected and evaluated by UV-VIS spectrophotometry. The wavelength to determine the concentration of vitamin C was previously evaluated by scanning wavelengths. The wavelength at which vitamin C shows greatest intensity is 264 nm, similar to the ones reported by other authors [58,59].

The encapsulation efficiency was calculated by:

(1) $$EE\;\left(\%\right)=\frac{M_{0}-M_{1}}{M_{0}}\times 100$$

where M_0_ is the mass of vitamin C acid weighed during the preparation of the emulsion and M_1_ is the mass of vitamin C acid determined in the filtered solution and washing waters.

### 4.6. Rheological Measurements

Pullulan bulk gelation time was determined by a series of frequency sweep tests in the linear viscoelastic region, previously determined by stress sweep tests at 1 Hz. The freshly prepared liquid mixture (pullulan 4% *w*/*w*, KOH 1.7% *w*/*w*, and STMP 1% *w*/*w*) was loaded into the rheometer. The mixture was loaded just after being prepared so that it gelled in the rheometer and thus monitored its evolution and the gelation point. A HAAKE-MARS III rheometer (ThermoElectron GmbH, Karlsruhe, Germany) was used with a serrated parallel plate measuring geometry (35 mm diameter, 1 mm gap) to avoid slippage. The temperature was controlled at 25.0 ± 0.1 °C. The instrument allowed data processing using HAAKE RheoWin Data Manager software Version 3.12 (ThermoElectron GmbH). The rheometer was provided with a cap with a small hole in the center to allow the passage of the rheometer sensor while keeping the sample covered in order to avoid evaporation of the mixture. Different wet gauze pads were placed inside the cap to favor a humidity-saturated environment and avoid evaporation of the sample. The rheometer was programmed to carry out different frequency sweeps at certain times in order to monitor the evolution of the pullulan gel formation and thus determine the needed time for the pullulan cross-linking extended to the whole bulk, i.e., the gelation time.

### 4.7. SEM Measurements

Characterization was performed at the Nanostructured Liquid Characterization Unit at the Institute of Advanced Chemistry of Catalonia (IQAC), which belongs to the Spanish National Research Council (CSIC) and is affiliated with the NANBIOSIS ICTS of the Biomedical Networking Center (CIBER-BBN).

First of all, the microcapsules were dispersed in water. The sample was deposited on the slide of the scanning electron microscope using a pasteur pipette. The sample was kept at room temperature until all the water was evaporated. Afterwards, the surface morphology of the microcapsules was observed using a TM 4000 Plus II Hitachi (Tokyo, Japan) SEM microscope.

### 4.8. Size Distribution

#### 4.8.1. Emulsion Droplet Mean Size Determination

The size of the emulsion droplets was determined by using optical microscopy (Olympus BX51TRF-6, Hsinchu County, Taiwan) with a digital camera (Olympus DP73, Tokyo, Japan) measuring 1000 droplets. The diameter measured in number-length was converted into the Brouckere diameter, d_4,3_, in order to compare the size distribution with that of the microcapsules obtained by laser difraction.

#### 4.8.2. Microcapsules Mean Size Determination

The microcapsules obtained were analyzed by laser diffraction with a Mastersizer 2000 (Malvern Instruments, MA, United Kingdom). The Brouckere mean diameter, related to volume distribution, was obtained from the microspheres size distribution. To obtain representative results of microsphere and particle size, analyses were performed in triplicate and expressed as mean values standard deviation (SD).

### 4.9. Stability Measurements

The stability of the emulsion was determined by studying the variation in the backscattering of the emulsion. A Turbiscan MA2000 instrument(Knutsford, United Kingdom) was used in order to record the backscattering of the emulsion. The backscattering detection method uses emulsion-scanning technology to monitor physical behavior in emulsions as a function of storage time.

### 4.10. Vitamin C Release Kinetics Experiments

In order to analyze the release of vitamin C, 1 g of microcapsules were placed in a dialysis bag (cut off 6000–8000 Da) and sealed. The bag was immersed in a beaker with 200 mL of buffer solution at different pH (pH = 2 and pH = 7). The system was stabilized at 25 °C and subjected to gentle magnetic stirring at 50 rpm. At time intervals, a sample of 2 mL was taken and analyzed by spectroscopy (Spectrophotometer UV-Vis 6305 Jenway, Leicestershire, United Kingdom). Previously, a wavelength scan with a vitamin C solution had shown that the peak of maximum concentration occurred at λ = 264 nm. Subsequently, 2 mL of the solution with the same pH was added to the corresponding system to maintain the same volume of liquid. The experimental data were fitted to the different models.

Zero-order model:

(2) $$\frac{M_{t}}{M_{\infty }}=k_{o}\cdot t$$

where $M_{t}/M_{\infty }$ is the fraction of vitamin C released in a time t, $k_{o}$ is the zero-order constant, and t indicates the elapsed time.

Korsmeyer–Peppas model:

(3) $$\frac{M_{t}}{M_{\infty }}=k_{P}\cdot t^{n}$$

where $M_{t}/M_{\infty }$ is the the fraction of vitamin C released in a time t, k_p_ is a constant, t indicates the elapsed time, and n is the release exponent. For 0 < n < 0.45, the release regime could be described as a hindered Fickian diffusion; for n ≈ 0.45, it could be described as Fickian diffusion; and for 0.45 < n < 1, an anomalous transport occurred [60,61].

### 4.11. Stability of Encapsulated Vitamin C

Iodometry was used to evaluate the vitamin C content of the capsules [54]. The blank consisted of the dispersed phase used to form the emulsion, but without the cross-linking agent, so that there was only pullulan and vitamin C. To evaluate vitamin C, a solution called Lugol reagen was used, formed by a mixture of iodine (I_2_) and potassium iodide (KI). To prepare it, first 3 g of KI was weighted and dissolved in 25 mL of milliQ water. Next, 0.35 g of I_2_ was added to the KI solution with a magnetic stirrer until complete dissolution.

Starch was used as an end-point indicator of the reaction. To prepare it, a beaker was filled with the necessary amount of milliQ water and heated until it boiled. The required amount of starch previously weighted was added and stirred until it was completely dissolved with a magnetic stirrer. Finally, the microcapsule sample to be evaluated was weighted, 50 mL of milliQ water was added, and it was homogenized with the Ultra-Turrax. Next, 2 mL of starch solution was added and the mixture was titrated with the previously prepared Lugol. At the moment that there is no more vitamin C to react with, the iodine will bind to the starch, providing the characteristic blue color, indicating that no more vitamin C is present in the sample.

## Figures and Tables

**Table 1:** Kinetic parameters of vitamin C release from pullulan microcapsules at different pHs.

	Zero-Order Model		Korsmeyer–Peppas		
	k	R^2^	k	n	R^2^
pH 1.8	0.4264	0.99283	0.1574	1.1809	0.98138
pH 7.0	0.3246	0.96062	9.0584	0.4044	0.96114

## Data Availability

Data are contained in this article. Extra data will be provided on request.

## Supplementary Materials

The following supporting information can be downloaded at: https://www.mdpi.com/article/10.3390/gels10060355/s1, Figure S1: Proposed mechanism of the cross-linking reaction of pullulan with STMP [30,31].
