# Supplementary figures and images for: Formation of Microcapsules of Pullulan by Emulsion Template Mechanism: Evaluation as Vitamin C Delivery Systems
Source: Gels. 2024 May 21;10(6):355. doi: 10.3390/gels10060355 (PMC11202853; doi:10.3390/gels10060355)

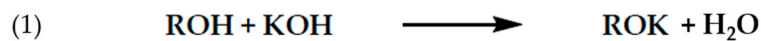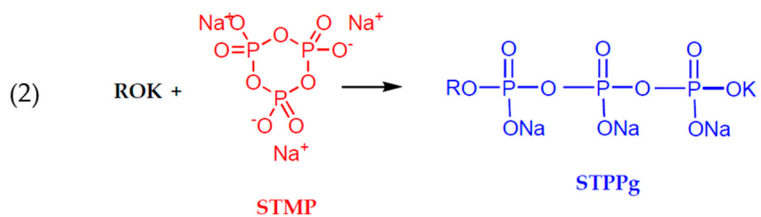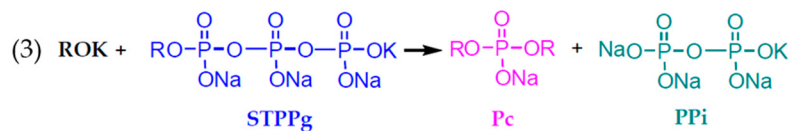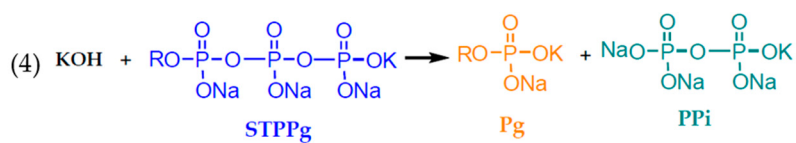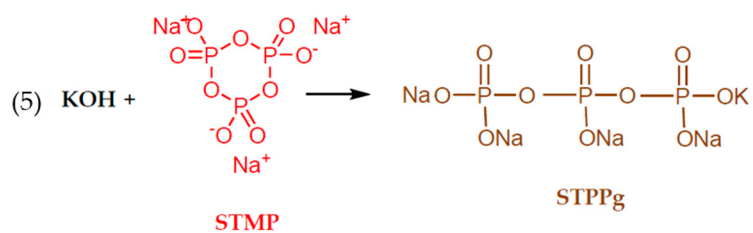

Figure S1. Proposed mechanism of the cross-linking reaction of pullulan with STMP [30-31].

Supplement: Supplementary file 1 [file gels-10-00355-s001.zip › gels-3012234-supplementary.pdf]
